# Quarantine Due to the COVID-19 Pandemic From the Perspective of Pediatric Patients With Type 1 Diabetes: A Web-Based Survey

**DOI:** 10.3389/fped.2020.00491

**Published:** 2020-07-31

**Authors:** Stefano Passanisi, Maria Pecoraro, Francesco Pira, Angela Alibrandi, Vittoria Donia, Paola Lonia, Giovanni Battista Pajno, Giuseppina Salzano, Fortunato Lombardo

**Affiliations:** ^1^Department of Human Pathology in Adult and Developmental Age “Gaetano Barresi”, University of Messina, Messina, Italy; ^2^Department of Ancient and Modern Civilizations, University of Messina, Messina, Italy; ^3^Unit of Statistical and Mathematical Sciences, Department of Economics, University of Messina, Messina, Italy

**Keywords:** children, coronavirus, lockdown, outbreak, management, resilience, technology

## Abstract

**Background:** A crucial aspect of the 2019 coronavirus disease (COVID-19) pandemic was the psychological impact on the population. Most countries issued restrictive laws to reduce community-based viral spread. Children and adolescents were forced to experience physical and social distancing. Subjects with chronic diseases, such as type 1 diabetes, were more vulnerable and at higher risk of developing psychological disorders.

**Methods:** We conducted a web-based survey to investigate the behavioral responses during quarantine due to the COVID-19 outbreak in a cohort of pediatric patients with type 1 diabetes. Data were collected on demographic and clinical characteristics, lifestyle changes, and the impact of COVID-19 on the management of diabetes.

**Results:** Two hundred four pediatric patients (aged 5–18 years) with type 1 diabetes completed the questionnaire. Interestingly, patients ≤12 years were significantly more influenced by the quarantine period in their approach to the disease than older patients.

**Conclusion:** Although quarantine was a stressful psychological condition, our results showed that most children and adolescents with type 1 diabetes developed high levels of resilience and excellent coping skills by using technology in a proper way.

## Introduction

Severe acute respiratory syndrome coronavirus 2 (SARS-CoV-2) infection, also known as 2019 Coronavirus disease (COVID-19), erupted in China on December 2019 and spread worldwide in very few months (1). Italy is currently one the most affected countries in Europe. The World Health Organization on March 11, 2020 declared COVID-19 a pandemic. The most direct and dramatic consequence of this pandemic is the high number of deaths due to SARS-CoV-2 infection. At the time of this publication 511,909 subjects died all over the world (2). A crucial, underestimated aspect is the heavy psychological impact on the population. Most countries issued strict governmental decrees that imposed self-isolation and social distancing in order to minimize community-based viral transmission. In Italy, the lock-down period lasted from March 9 to May 3, 2020 for a total of 55 days. Hospital shut their outpatient services, deferring all “non-urgent” healthcare activities (3). These restrictive measures led to a gradual reduction of new cases of infection (4). However, people were forced to radically change their daily lifestyles and were at high risk of developing feelings of panic, anxiety, depression, and sometimes even dread (5). The psychological aspects of the COVID-19 pandemic also influenced the pediatric population due to the strong experience of physical and social isolation (6). In addition, children and adolescents suffering from type 1 diabetes (T1D) were unable to comply with scheduled outpatient follow-up visits and were also forced to modify the approach to the management of their chronic disease.

Aim of this study was to investigate the behavioral responses during the quarantine due to the COVID-19 pandemic in a cohort of Italian pediatric patients with T1D.

## Materials and Methods

From April 15 to May 1, 2020 we conducted a cross-sectional survey based on an on-line questionnaire. We enrolled 204 children and adolescents (aged 5–18 years) diagnosed with T1D for at least 3 months, and followed-up at our Pediatric Diabetes Centre in Messina. The online link for the questionnaire was sent to one of the parents and to the patient itself if over 12 years, and they were encouraged to fill it out together. Written informed consent through on-line form was obtained from patients' parents. The study was conducted in accordance with the Helsinki Declaration. The questionnaire included fourteen questions focusing on patients' demographic and clinical characteristics (e.g., age, gender, diabetes duration, insulin regimen, type of glucose monitoring), lifestyle changes during the quarantine period, and the impact of the lock-down on the management of diabetes. Finally, the participants were asked to quantify how much the quarantine influenced their approach to the disease according to the following four response levels: no influence, poor influence, relevant influence, extreme influence. Furthermore, the results of questionnaire were evaluated between two age groups (5–12 and 13–18 years).

An English translation of the full Italian questionnaire is available as Supplementary Material.

Demographic and clinical patients' characteristics and results of questionnaire were statistically analyzed. The numerical data were expressed as mean and standard deviation, and the categorical variables as absolute frequencies and percentages. In order to compare patients less or more than 12 years we applied Mann-Whitney test for numerical parameters and Chi Square test for categorical variables. The influence of patients' clinical and demographical characteristics (i.e., gender, age group, diabetes duration, type of insulin treatment and glucose monitoring system) on the perception of the impact of quarantine in the approach to T1D management was assessed by the cumulative proportional odds model. A *P*-value smaller than 0.050 was considered to be statistically significant.

## Results

Mean age of our study population was 12.2 ± 3.6 years, with a prevalence of male gender (57.8%). Mean duration of T1D was 5.2 ± 3.7 years. Interestingly, most patients (72.5%) modified their sleep-wake rhythm. The use of technology was predominant both for recreational activities (communications, games, videos) and for educational purposes (scholar, musical and sportive activities). Less than 5% declared they did not use technology during this quarantine period. The average time spent on technology was mostly 4–6 h a day for educational purposes, and 1–3 h a day for recreational activities. Almost all the subjects (96.1%) took advantage of this period to acquire new skills, such as cooking, do it yourself activities learning, and reading books (13.7%). Regarding the dietary lifestyle, more than half of patients (56.9%) did not change their eating habits during the lock-down period. Fifty-four subjects (26.5%) increased carbohydrate consumption, 16 (7.8%) and 18 (8.8%) patients ate a large amount of fat and protein, respectively. Despite Italian governmental decrees prohibited outdoor sports, 63.3% of our patients regularly practiced physical activity at home. Particularly, 27.9% of patients spent from 1 to 3 h a week for physical activities, 14.7% of subjects spent less than an hour a week to do sports, and 20.6% of patients declared that physical activities kept them busy at least 4 h a week. Regarding the daily glucose monitoring, 33.8% of patients reported that it was more intensive during the quarantine period. Instead, 18.6% of the study participants paid less attention to their glycemic levels, and 47.5% of patients did not report differences from the pre-quarantine period. Interestingly, almost half of the patients (49%) did not need to contact the Diabetes team for advice on managing their disease. The most common used communication modality between patients and diabetes specialists was text messages, followed by e-mail messages and phone calls. None of the surveyed patients needed to be acutely evaluated during the lockdown period for diabetes-related acute complications (i.e., severe hypoglycemia, diabetic ketoacidosis). Finally, 45.6% of patients reported that the quarantine was an additional heavy burden on their perspective of the disease. Among these patients, 36.3% reported a relevant impact and 9.3% referred an extreme impact. On the contrary, 16.2% of the study participants declared that the quarantine did not affect their psychological and practical approach to diabetes, and 38.2% of patients partially suffered the consequence caused by the lock-down measures (Table 1).

**Table 1:** Overview of the results of web-based survey.

N^°^	204
Age (years)	12.2 ± 3.6
Female (%)	86 (42.2%)
Duration of diabetes (years)	5.2 ± 3.7
**Type of treatment**	
Multiple daily injection	84 (41.2%)
Continuous subcutaneous insulin infusion	120 (58.8%)
**Glucose monitoring system**	
Self-monitoring blood glucose	56 (27.5%)
Continuous glucose monitoring or Flash glucose monitoring	148 (72.5%)
**Modification of sleep-weak rhythm**	
Yes	147 (72.5%)
No	55 (27.5%)
**New skills acquired**	
Cooking	54 (26.5%)
Reading books	28 (13.7%)
Do it yourself activities	35 (17.2%)
Art of music (singing, play an instrument)	18 (8.8%)
Housecleaning	12 (5.9%)
Others	49 (24%)
None	8 (3.9%)
**Time spent on technology for recreational activities**	
<1 h a day	36 (17.6%)
1–3 h a day	73 (35.8%)
4–6 h a day	43 (21.1%)
>6 h a day	44 (21.6%)
Not used	8 (3.9%)
**Time spent on technology for educational purposes**	
<1 h a day	14 (6.8%)
1–3 h a day	56 (27.5%)
4–6 h a day	69 (33.8%)
>6 h a day	59 (28.9%)
Not used	6 (2.9%)
**Variations in eating habits**	
Increased carbohydrate consumption	54 (26.5%)
Increased fat consumption	16 (7.8%)
Increased protein consumption	18 (8.8%)
No differences	116 (56.9%)
**Time spent on physical activity at home**	
<1 h a week	30 (14.7%)
1–3 h a week	57 (27.9%)
4–6 h a week	27 (13.2%)
>6 h a week	15 (7.4%)
Not practiced	75 (36.7%)
**Variations in the approach to glucose monitoring**	
More intensive	69 (33.8%)
Less intensive	38 (18.6%)
No differences	97 (47.5%)
**How to contact the diabetes team**	
Email messages	35 (17.2%)
Phone calls	18 (8.8%)
Text messages	51 (25%)
No contact	100 (49%)
**How much the quarantine influenced the approach to the disease**	
No influence	33 (16.2%)
Poor influence	78 (38.2%)
Relevant influence	74 (36.3%)
Extreme influence	19 (9.3%)

When the two age groups were compared (Table 2), a significant difference was found in the duration of T1D (*P* < 0.001). Older patients reported they spent more hours for physical activities than younger subjects (*P* < 0.001). On the contrary, patients aged ≤12 years measured glucose levels more frequently (*P* = 0.028). Interestingly, they were significantly more influenced by the quarantine period in their approach to the disease than older patients (*P* = 0.017). No further differences were found between the two groups. The cumulative proportional odds model showed that the younger age was the only factor that was significantly related to the different perception of the influence of quarantine in the approach to T1D management in our study population (*P* = 0.007) (Table 3).

**Table 2:** Differences regarding the management of type 1 diabetes between patients <12 years group and patients ≥12 years group.

**Variables**	**Patients ≤12 years**	**Patients >12 years**	***P*-value**
**Gender**			0.571
Male	55.9%	59.8%	
Female	44.1%	40.2%	
**Duration of T1D (years)**	3.8 ± 2.6	6.6 ± 4.1	**<0.001**
**Type of treatment**			0.155
Multiple daily injection	46.1%	36.3%	
Continuous subcutaneous insulin infusion	53.9%	63.7%	
**Glucose monitoring system**			0.120
Self-monitoring blood glucose	22.5%	32.4%	
Continuous glucose monitoring or flash glucose monitoring	77.5%	67.6%	
**Variations in the approach to glucose monitoring**			**0.028**
More intensive	39.2%	28.4%	
Less intensive	22.5%	14.7%	
No differences	38.2%	56.9%	
**Time spent on physical activity at home**			**0.001**
<1 h a week	18.6%	10.8%	
1–3 h a week	22.5%	33.3%	
4–6 h a week	7.8%	18.6%	
>6 h a week	3.9%	10.8%	
Not practiced	47.1%	26.5%	
**Variations in eating habits**			0.195
Increased carbohydrate consumption	21.5%	25.5%	
Increased fat consumption	8.8%	6.9%	
Increased protein consumption	4.9%	8.8%	
No differences	64.7%	58.8%	
**How to contact the diabetes team**			0.271
Email messages/Phone calls/Text messages	54.9%	47.1%	
No contact	45.1%	52.9%	
**The influence of quarantine on the approach to diabetes**			**0.017**
No influence	12.7%	19.6%	
Poor influence	31.4%	45.1%	
Relevant influence	42.2%	30.4%	
Extreme influence	13.7%	4.9%	

*The bold values mean that the result is statistically significant*.

**Table 3:** The relationship between patients' clinical and demographical characteristics and the perception of the influence of quarantine in their approach to type 1 diabetes.

**Variables**	**Coefficient**	**95%C.I**.	***P*-value**
Constant 1^*^	−2.141	−2.949; −1.332	**<0.001**
Constant 2^*^	−0.260	−1.006; 0.485	0.494
Constant 3^*^	1.910	1.087; 2.733	**<0.001**
Gender	−0.162	−0.684; 0.360	0.543
Age group	−0.779	−1.343; −0.215	**0.007**
Diabetes duration	−0.005	−0.083; 0.072	0.898
Type of treatment	0.102	−0.482; 0.687	0.731
Glucose monitoring systems	0.025	−0.608; 0.658	0.938

* *The cumulative proportional odds model uses a number of constants equal to the number of the variables included in the outcome (response levels regarding the influence of the lock-down period on the approach to the disease) minus 1*.

*The bold values mean that the result is statistically significant*.

## Discussion

T1D is a metabolic disease characterized by the progressive decline of pancreatic β cells functions leading to relative or absolute insulin deficiency (7). T1D is one of the most frequent autoimmune disorders in the pediatric population, and its incidence and prevalence are increasing worldwide (8). It is estimated that about 18,000 children and adolescents are currently affected by T1D in Italy (9). Evidence shows that a large number of pediatric patients, especially diabetic adolescents, experience disease-related impairment of quality of life and may develop depression, anxiety and other psychological states (10, 11). Psychological and behavioral disorders in T1D pediatric patients have been demonstrated to be related to negative health outcomes, such as brittle glycemic control and high risk of acute and chronic complications (12).

Although no data are available on the exact number of symptomatic and asymptomatic subjects positive for COVID-19 in the pediatric age, children appear to be less infected and if infected develop milder clinical pictures due to SARS-CoV-2 infection (13). A descriptive cases series of 130 Italian children with a confirmed diagnosis of COVID-19 reported that only 8.5% of these had a severe disease, and 6.9% had a critical presentation (14). Another Italian study involving a cohort of 100 hospitalized children affected by COVID-19 demonstrated that only nine patients needed respiratory support (15). Children and adolescents have been impacted psychologically experiencing various behavioral issues (6). The risk of acute stress disorder, adjustment disorder and grief in children who are quarantined during pandemic diseases had already been well-described (16). Furthermore, people suffering from a chronic disorder, such as T1D, are more vulnerable and at higher risk for developing dangerous feelings, such as uncertainties, distraction, irritability, and fear.

However, our results showed that most of the study participants reacted reassuringly to this new social condition as demonstrated by the responses regarding the management of the disease. In fact, the majority of children and adolescents with T1D were able to comply with the landmarks of the management of diabetes (i.e., healthy and balanced diet, regular physical activity and careful glucose monitoring). More than half of patients reported having avoided overeating during this quarantine period. We suppose that abstention from school and peer relationships out of school, has helped to maintain a healthy diet since numerous extra meals disappeared. Despite the lock-down measures, almost two third of our patients regularly have engaged in physical activity. Regular physical exercise is known to help subjects with T1D achieve good glycemic control, as well as improve lipid profile, body composition and well-being (17). Regarding the daily glucose monitoring, our results showed that the quarantine period negatively influenced only 18.6% of patients who reported a less intensive control of their glycemic values.

These findings suggest that pediatric patients with T1D developed functional “empowerment” as a response to the social emergency. Furthermore, they showed greater awareness of their disease and excellent coping skills by using technology in a proper way (18). Technology has played a crucial role in the quarantine approach for T1D children. In fact, technological medical devices (e.g., insulin pump, glucose sensor) have facilitated the management of the disease, while other technological tools, such as smartphones, tablets, and personal computers have preserved the “social dimension” even during the lock-down. Thanks to the availability of technology, children and adolescents have been able to continue school learning and to ensure social networks by minimizing negative emotions related to the social isolation.

Although physical freedom has been limited by the lock-down, new individual resources have been emerged due to personal and familiar factors, but also thanks to the school system and friendly system which have been kept active through technological tools (19).

Interestingly, patients >12 years reported having practiced indoor physical activities more regularly than younger patients and, above all, they were significantly less affected by the quarantine period in their approach to the disease. Adolescence is a well-known, high-risk time period for all young people who experience rebellion and lawlessness. It is widely demonstrated that the adolescent population with T1D is at high risk of poor clinical outcomes (20). However, our findings highlight that adolescence is also a crucial phase of life for the individual since it allows the achievement of a satisfactory level of interior maturity and new personal skills (21). Instead, patients ≤12 years were mainly affected by the quarantine as they are still in need for reassurance and parental care, and appear uncertain in the management of the disease because of recent diagnosis and/or poor autonomy. Therefore, our finding that this age group monitored glycemic levels more intensely could also be explained by the more rigorous parental control in the various aspects of T1D management.

In conclusion, the present study demonstrated that children and adolescents with T1D showed high levels of resilience. Although quarantine was a stressful psychological condition, pediatric patients were able to overcome their limits to reach new interior resources and strengthened self-awareness.

## Data Availability Statement

All datasets presented in this study are included in the article/Supplementary Material.

## Ethics Statement

Ethical review and approval was not required for the study on human participants in accordance with the local legislation and institutional requirements. Written informed consent to participate in this study was provided by the participants' legal guardian/next of kin.

## Author Contributions

FL conceived the designed study and approved the final version of the manuscript. SP drafted and wrote the paper. MP and FP analyzed the results and helped to write the paper. VD and PL sent the questionnaire link to the patients and collected the results. AA realized the statistical analysis. GP and GS contributed to the discussion and reviewed the paper. All authors contributed to the article and approved the submitted version.

## Conflict of Interest

The authors declare that the research was conducted in the absence of any commercial or financial relationships that could be construed as a potential conflict of interest. The handling editor declared a past co-authorship with several of the authors FL and GS.
