# Supplementary material for: Quarantine Due to the COVID-19 Pandemic From the Perspective of Pediatric Patients With Type 1 Diabetes: A Web-Based Survey
Source: Front Pediatr. 2020 Jul 31;8:491. doi: 10.3389/fped.2020.00491 (PMC7411347; doi:10.3389/fped.2020.00491)
Supplement: Supplementary file 1 [file Data_Sheet_1.docx]

**Questionnaire: the experience of quarantine due to COVID-19 in pediatric patients with type 1 diabetes**

1. How old are you?

2. Gender

1. Male
2. Female

3. How many years have you had type 1 diabetes?

4. What is your type of treatment regimen?

1. Multiple daily insulin injections
2. Continuous subcutaneous insulin infusion

5. Which glucose monitoring system do you use?

1. Self-monitoring blood glucose
2. Continuous (or flash) glucose monitoring

6. Have you observed any changes in your approach to glucose monitoring?

1. More intensive
2. Less intensive
3. No differences

7. Have you observed any variations of your eating habits?

1. Increased carbohydrate consumption
2. Increased fat consumption
3. Increased protein consumption
4. No differences

8. Have you practiced physical activity at home?

1. <1 hour a week
2. 1-3 hours a week
3. 4-6 hours a week
4. >6 hours a week
5. No physical activity

9. How much time did you spend on technology for educational purposes (scholar, musical and sportive activities)?

1. <1 hour a day
2. 1-3 hours a day
3. 4-6 hours a day
4. >6 hours a day
5. Not used for this purpose

10. How much time did you spend on technology for recreational purposes (communications, games, videos)?

1. <1 hour a day
2. 1-3 hours a day
3. 4-6 hours a day
4. >6 hours a day
5. Not used for this purpose

11. Have you observed changes in your sleep-weak rhythm?

1. Yes
2. No

12. Which new skills did you acquire during this period?

1. Art of music (singing, musical instrumental)
2. Cooking
3. Do it yourself activities
4. Housecleaning
5. Reading books
6. Others
7. No new skills acquired

13. How did you contact your Diabetes team?

1. Email messages
2. Phone calls
3. Text messages
4. None contact

14. How much do you feel that quarantine influences the approach to your disease?

1. No influence
2. Poor influence
3. Relevant influence
4. Extreme influence
